# *Drosophila* Cbp53E Regulates Axon Growth at the Neuromuscular Junction

**DOI:** 10.1371/journal.pone.0132636

**Published:** 2015-07-13

**Authors:** Kimberly R. Hagel, Jane Beriont, Charles R. Tessier

**Affiliations:** 1 Department of Biological Sciences, University of Notre Dame, Notre Dame, Indiana, United States of America; 2 Department of Medical and Molecular Genetics, Indiana University School of Medicine-South Bend, South Bend, Indiana, United States of America; University of Sydney, AUSTRALIA

## Abstract

Calcium is a primary second messenger in all cells that functions in processes ranging from cellular proliferation to synaptic transmission. Proper regulation of calcium is achieved through numerous mechanisms involving channels, sensors, and buffers notably containing one or more EF-hand calcium binding domains. The *Drosophila* genome encodes only a single 6 EF-hand domain containing protein, Cbp53E, which is likely the prototypic member of a small family of related mammalian proteins that act as calcium buffers and calcium sensors. Like the mammalian homologs, Cbp53E is broadly though discretely expressed throughout the nervous system. Despite the importance of calcium in neuronal function and growth, nothing is known about Cbp53E’s function in neuronal development. To address this deficiency, we generated novel null alleles of *Drosophila* Cbp53E and examined neuronal development at the well-characterized larval neuromuscular junction. Loss of Cbp53E resulted in increases in axonal branching at both peptidergic and glutamatergic neuronal terminals. This overgrowth could be completely rescued by expression of exogenous Cbp53E. Overexpression of Cbp53E, however, only affected the growth of peptidergic neuronal processes. These findings indicate that Cbp53E plays a significant role in neuronal growth and suggest that it may function in both local synaptic and global cellular mechanisms.

## Introduction

Calcium is a major second messenger in all cells and is vital for cellular function. Given this importance, all aspects of calcium homeostasis including influx, efflux, intracellular mobilization, and storage are tightly controlled processes[1]. Excitable cells, such as neurons, are particularly susceptible to changes in calcium dynamics, as calcium is required for synaptic vesicle fusion and neurotransmitter release [2]. Even small defects in calcium homeostasis can affect synaptic transmission and result in profound consequences ranging from individual neuronal dysfunction to aberrant circuit formation and altered behavior [3–5]. Therefore, understanding how calcium is regulated is critical for understanding neuronal development and function. An important class of calcium regulators that function in neural development are the calcium buffering proteins.

Proteins that function as calcium buffers are thought to bind and sequester intracellular calcium ([Ca^2+^]_i_) in order to modulate its spatiotemporal availability as a second messenger signaling molecule[6]. There are many vertebrate calcium buffering proteins including parvalbumin, calretinin, calbindin-D9k and calbindin-D28k. Each of these proteins contains one or more EF-hand binding domains, a motif capable of binding calcium ions, though not all EF-hand domains may be active under physiological conditions[7]. Moreover, the affinity of these domains for calcium may be different across different buffering proteins. Thus, some calcium buffering proteins are considered slow buffers and others fast buffers, depending on several factors including their affinity for calcium, binding kinetics, and their cytoplasmic mobility[8]. This distinction is particularly important in neurons, which have both pre- and postsynaptic compartments capable of responding to membrane depolarization through multiple calcium influx and calcium mobilizations pathways[9]. Thus, different aspects of calcium homeostasis may be regulated by different buffering proteins.

The two major vertebrate calcium buffers, calbindin-D28k (CB) and calretinin (CR), share 58% identity to each other and are members of a small family of 6 EF-hand domain containing proteins that are classically characterized as fast calcium buffers[10, 11]. A third protein, secretagogin shares significant homology to these buffers, but has only been characterized as a calcium sensor [12, 13]. Calcium sensors typically bind target proteins to affect signaling in response to changes in [Ca^2+^]_i_. Indeed, CB and CR were once thought to function exclusively as buffers, but now both have been shown to bind effector proteins under different calcium-dependent situations [14, 15]. CB and CR are expressed throughout the brain in discrete, mostly exclusive patterns, predominantly in the cerebellum, hippocampus, and cortex [16–19]. Each protein affects Purkinje cell development, a function responsible for motor coordination defects found in CB and CR knockout mice [20–22]. At the cellular level, CB mitigates calcium influx transient amplitude in dendrites, while CR can affect the timing of certain functional spikes [21–23]. Neuronal function is also impaired in the hippocampus where loss of CB or CR leads to defects in long-term potentiation as well as animal failures in spatial learning tests [24, 25]. In addition to roles in synaptic plasticity, these buffers seem to have neuroprotective roles in neurons involved in Parkinson’s disease (PD) and Alzheimer’s disease (AD)[26–30]. Interestingly, the neuroprotective functions may be in part due to a direct interaction with target binding proteins which can affect downstream signaling cascades[31]. Therefore, CB and CR are multifunctional proteins that regulate calcium homeostasis to control neuronal development and function.

*Drosophila* are now routinely being used as models for understanding both normal neuronal development and disease states such as PD and AD[32]. However, little work has been done to understand the role of calcium buffers in this well-established model organism. The *Drosophila* genome encodes only a single 6 EF-hand domain containing protein, Cbp53E [33]. Cbp53E shares nearly the same homology to all 3 members of the mammalian family, having 45%, 46%, and 45% protein sequence identity with CB, CR, and secretagogin respectively. Although Cbp53E was cloned over two decades ago, virtually nothing is known about its function [33, 34]. We have generated two novel null alleles of the *Drosophila* Cbp53E gene in order to determine its role in neuronal development. We describe Cbp53E-dependent effects on axon growth which seem to implicate both local synaptic and global cellular functions for this highly conserved protein.

## Materials and Methods

### Fly stocks and genetics

The control strain was *w* ^*1118*^. Cbp53E mutants are: *Cbp53E* ^*Mi22*^, *Cbp53E* ^*Mi41*^, and *Cbp53E* ^*Mi2*^. The 2^nd^ chromosome deficiency is Df(2R)ED2751. Animals used in rescue experiments are: elav-GAL4/+ (control) and elav-GAL4/+; *Cbp53E* ^*Mi22*^ and elav-GAL4/+; UAS-Cbp53E/+ and elav-GAL4/+; *Cbp53E* ^*Mi22*^; UAS-Cbp53E/+ (rescue), 24B-GAL4/+ (control) and *Cbp53E* ^*Mi22*^; 24B-GAL4 and 24B-GAL4/UAS-Cbp53E and *Cbp53E* ^*Mi22*^; 24B-GAL4/UAS-Cbp53E (rescue). Flies used for motor neuron analysis were: UAS-mCD8GFP/+;D42-GAL4/+ and flies used for 24B-GAL4 analysis were: UAS-mCD8GFP/+;24B-GAL4/+.

### Cbp53E mutant allele generation

All lines used in mutant generation were acquired from the Bloomington *Drosophila* Stock Center. The transposon insertion Mi{ET1}Cbp53E^MB00150^ was mobilized by crossing it to a heat shock inducible Minos transposase P{hsILMiT}2.4 using standard techniques[35]. Flies were screened for loss of GFP expression present on the transposon containing chromosome and subsequently outcrossed to remove the transposase. Individual progeny were then crossed to a second chromosome deficiency line. To determine the effect of transposon mobilization on Cbp53E expression, brains from the progeny were stained for Cbp53E by immunohistochemistry. Mutant lines *Cbp53E* ^*Mi22*^ and *Cbp53E* ^*Mi41*^ were then sequenced using PCR primers around the transposon insertion site to produce a PCR product approximately 2kb in length. The primers used were:

forward: 5′-CCCATTTCCCATTGCATTTCTAAGAGC-3′;

reverse: 5′-GCCAGTGTTAGCAGAATCATGGTCAGT-3′. Sequence analysis revealed that Cbp53E sequences remained intact around the insertion, but that approximately 7.6kb of the 3′-most portion of the transposon had been removed from each mutant line.

### Transgenic generation

RNA was extracted from *w* ^*1118*^ flies using Trizol (Invitrogen) and then used to make cDNA by Superscript III Reverse Transcriptase (Invitrogen) as previously[36]. The following cloning primers were used to amplify Cbp53E cDNA from the total cDNA with *Not1* and *KpnI* restriction sites:

forward: 5′-AAATTTGCGGCCGCTTCTCCAAAGATA-3′;

reverse: 5′-AATGGTACCTTTTGGAGTGCTGACCCC-3′. The resultant PCR product encompassed the entire Cbp53E mRNA including untranslated regions. The PCR product was purified and digested with *Not1* and *Kpn1* and ligated into a similarly digested pUAS-T vector (*Drosophila* Genomics Resource Center). The recombined plasmid was purified and microinjected into *w* ^*1118*^ *Drosophila* embryos by Genetic Services INC (Waltham MA). Transformants were identified and mapped to chromosomes using standard techniques. UAS-Cbp53E was balanced over TM6 for stock maintenance.

### Immunohistochemistry

Adult *Drosophila* brains were dissected in phosphate buffered saline (PBS) and collected in PBS; 1% bovine serum albumin (BSA) before fixing in a PBS; 4% formaldehyde; 4% sucrose solution for 30 minutes at 25°C. Similarly, wandering third instar larvae were dissected in PBS and fixed as above. Brains and larval preparations where then washed with PBS; 1% BSA; 0.2% Triton-X 100 three times for 30 minutes each at 25°C. All samples were then incubated in primary antibody for 14–16 hours at 4°C, washed three times in wash solution for 30 minutes each at 25°C, and then incubated in secondary antibody for 1 hour. Finally, samples were washed three times for 45 minutes each before mounting in Fluoromount G (eBioscience, Inc.). Primary antibodies used: mouse anti-Cbp53E (1:50, DCB32(pok13), Developmental Studies Hybridoma Bank)[37], guinea pig anti-insulin (1:50, ab7842, Abcam), rabbit anti-horseradish peroxidase (1:200, Jackson Laboratories), mouse anti-Discs Large (1:200, 4F3, Developmental Studies Hybridoma Bank). Secondary antibodies used (all at 1:2000 dilutions): AlexaFluor 647 goat anti-mouse (Invitrogen), AlexaFluor 488 goat anti-rabbit (Invitrogen), AlexaFluor 488 goat anti-mouse (Jackson Laboratories), and Cy3 goat anti-guinea pig (Invitrogen).

### Quantitative RT-PCR

cDNA was generated as described above from heads of mutant and control animals. POWER SybrGreen (Applied Biosystems) was then used according to the manufacturer’s instructions to perform quantitative PCR for Cbp53E expression using a BioRad CFX Connect Real Time System. Primers for Cbp53E detection were:

forward: 5′-GCCAACAAGGACGGACGTCTGCAGTT-3′;

reverse: 5′-GGCATCATAGTCGTCCTTCTTGACCAA-3′. GAPDH2 was used as the reference control with the following primers:

forward: 5′-CCGATGCGACCAAATCCATTGATA-3′;

reverse: 5′-CGCTCAAAATTTCTCAGCCATCAC-3′. Cycling parameters were: 96°C-2min, (94°C-30sec, 60°C-30sec, 72°C-30sec) X40, 72°C-1min. The ΔΔCt method was used to calculate relative fold changes of Cbp53E expression in mutant versus control animals.

### Confocal microscopy

Z-stacks of segment 3, muscle 4, muscle 6/7 and muscle 12/13 neuromuscular junctions and whole brains were acquired on a Zeiss LSM 710 confocal microscope under 10X (1.3na), 40X (1.3na) or 63X (1.4na) magnification. ImageJ and Zeiss ZEN software were used to analyze maximum intensity projections or single slice sections of Cbp53E expression or axonal elaborations. Branches were defined as at least two boutons connected to the NMJ arbor as before [38].

### Statistical Analyses

ANOVAs were performed on all data sets followed by a post-hoc Newman-Keuls multiple comparisons test using GraphPad Prism software. All error bars are given as Standard Error Mean. Significance definitions are *0.01<p<0.05, **0.001<p<0.01, ***0.001<p.

## Results

**Fig 1:**
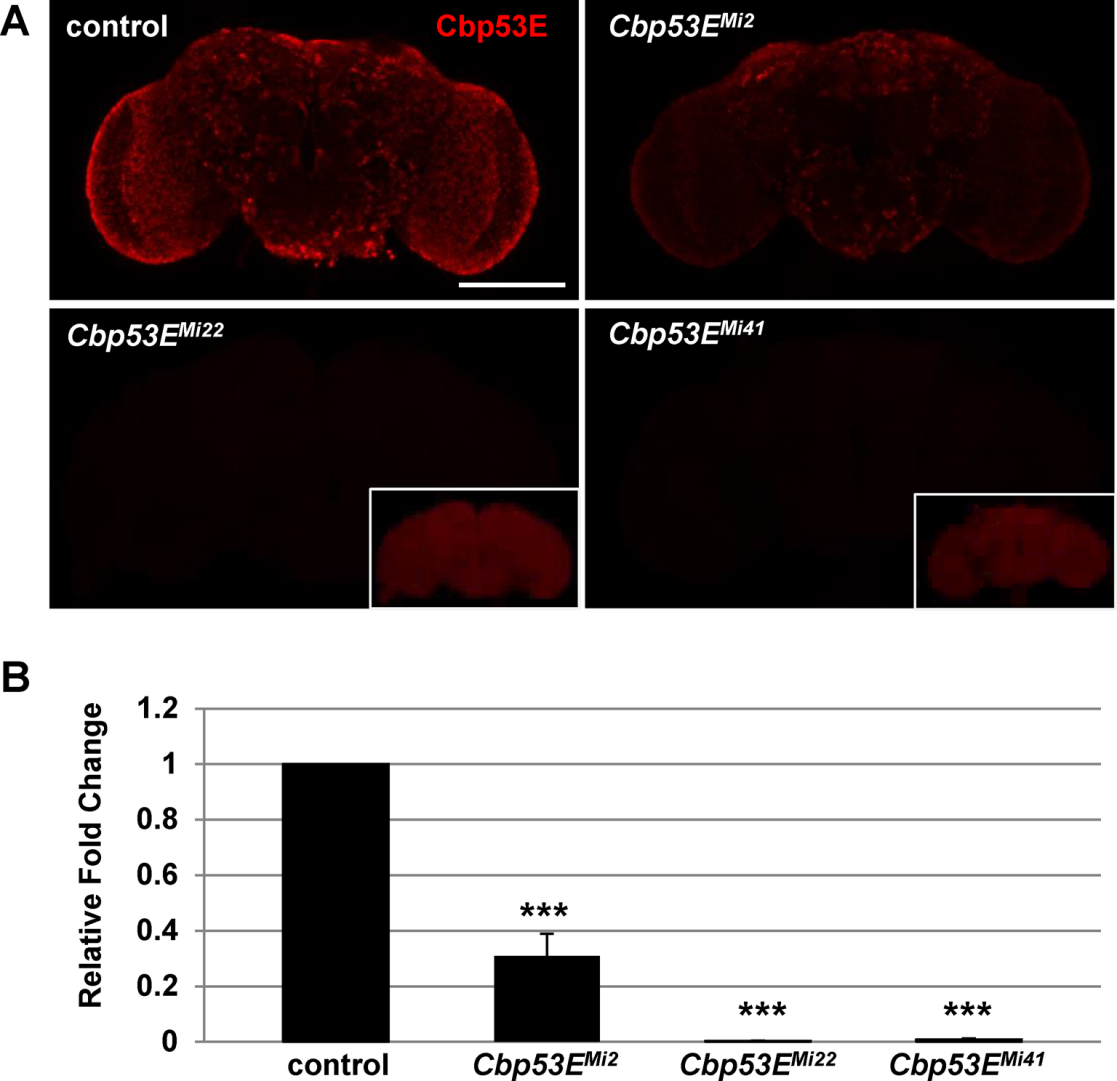
Generation of novel loss of function Cbp53E alleles. A) A Minos transposable element in the 3′ portion of the CBP53E locus was mobilized to generate 3 new alleles with reduced Cbp53E expression in the adult brain. *Cbp53E* ^*Mi2*^ shows residual Cbp53E, while alleles *Cbp53E* ^*Mi22*^ and *Cbp53E* ^*Mi41*^ are complete nulls. Insets show the same image with increased detector gain settings. Scale bar is 100μm. B) Quantitative RT-PCR of Cbp53E mRNA levels normalized to GAPDH2 from the indicated genotypes and reported as the relative fold change to control levels. *** p<0.001.

Cbp53E is the *Drosophila* homolog of the mammalian CB/CR family of 6 EF-hand domain containing proteins[33]. Though neuronal expression of Cbp53E was reported long ago, nothing is known about how this protein affects neuronal development [33, 34]. In order to begin to parse the function of Cbp53E, we attempted to identify null Cbp53E alleles by screening existing transposable elements within this locus using anti-Cbp53E antibodies [37]. All insertion lines retained a small amount of residual Cbp53E expression as measured by whole brain immunohistochemistry (data not shown). We therefore identified the Minos transposable element MiET1 located in the 3′ portion of the Cbp53E gene and mobilized it through standard techniques to generate new mutant alleles [35]. Three alleles with reduced Cbp53E expression were identified through this process. Cbp53E is normally expressed throughout the adult brain; however the *Cbp53E* ^*Mi2*^ allele exhibited substantially reduced expression while staining in the *Cbp53E* ^*Mi22*^ and *Cbp53E* ^*Mi41*^ alleles was completely absent (Fig 1A). These findings were confirmed at the RNA level as quantitative RT-PCR revealed essentially no Cbp53E mRNA present in *Cbp53E* ^*Mi22*^ and *Cbp53E* ^*Mi41*^ heads while *Cbp53E* ^*Mi2*^ animals only showed an approximately 70% reduction in Cbp53E mRNA relative to controls (Fig 1B). Thus, *Cbp53E* ^*Mi22*^ and *Cbp53E* ^*Mi41*^ were considered null alleles, since each completely lacked Cbp53E expression. None of the alleles exhibited any overt developmental phenotypes, and all were homozygous viable. Preliminary experiments could not identify any molecular or structural defects in *Cbp53E* ^*Mi2*^, and since it retained some Cbp53E expression, only the two true null alleles were considered for further experimentation.

### *Drosophila* Cbp53E is expressed at the neuromuscular junction

**Fig 2:**
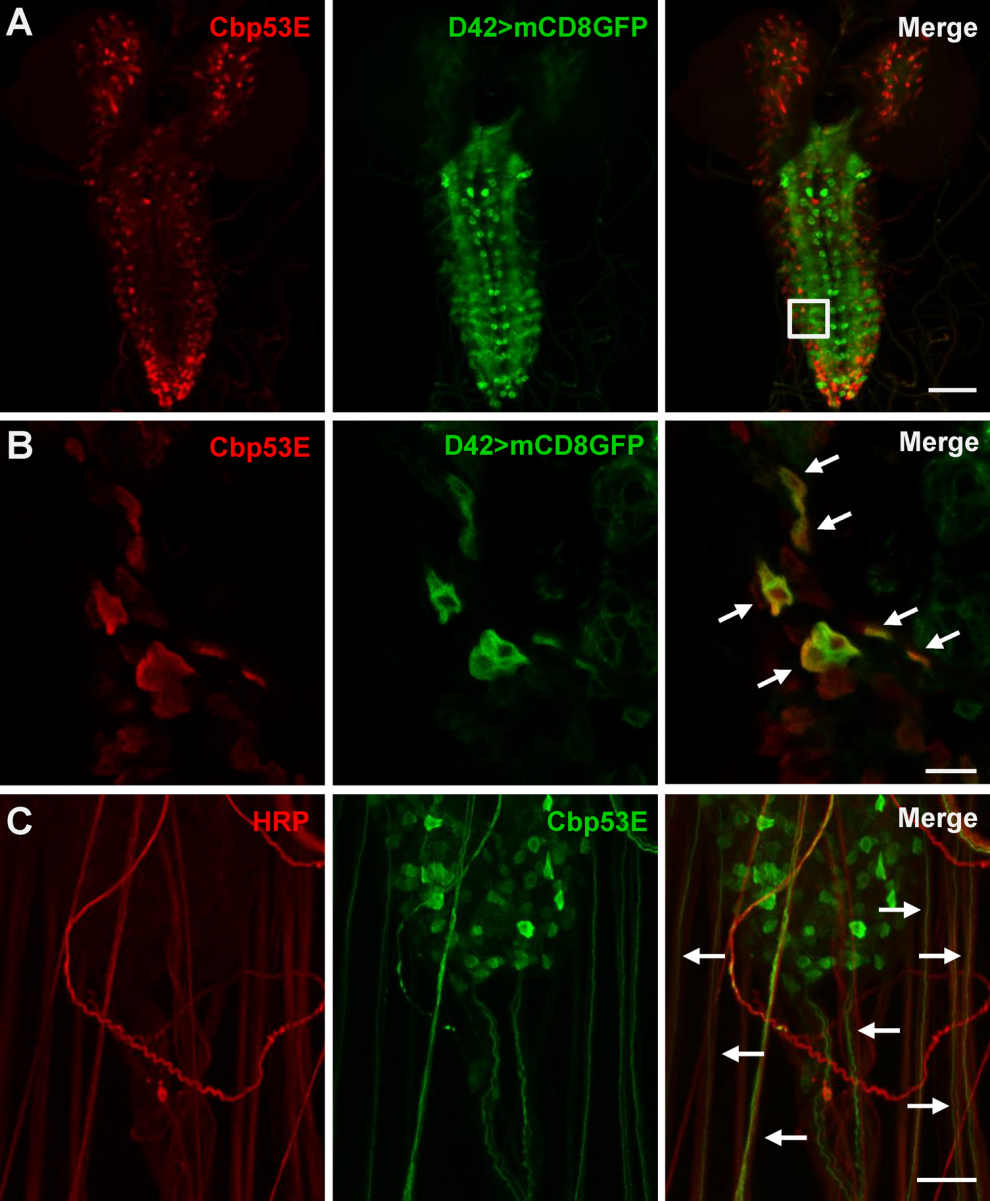
Expression of Cbp53E in the larval brain. A) Representative maximum intensity projection of a third instar larval brain from animals expressing UAS-mCD8GFP (green) from a D42-GAL4 motor neuron specific driver. Anti-Cbp53E staining (red) shows discrete expression patterns. Scale bar is 50μm. B) A single slice from the magnified region outlined in the merge panel in (A). Significant colocalization of anti-Cbp53E (red) and GFP (green) is seen in many cells (arrows). Scale bar is 10μm. C) Strong Cbp53E expression (green) can be seen in nerve fibers labeled with anti-HRP (red) as they exit the larval central nervous system (arrows). Scale bar is 20μm.

The *Drosophila* larval system has been extensively used to characterize the effects of genetic mutations on neuronal growth [39]. Since Cbp53E expression has mostly been characterized in adult animals, we used immunohistochemistry to determine where Cbp53E was localized in larval neuronal tissues. In the third instar larval brain, Cbp53E was expressed in a broad but discrete pattern but was completely absent from *Cbp53E* ^*Mi22*^ null animals (Fig 2A and S1 Fig). In order to characterize some of this pattern, we specifically identified motor neurons by expressing GFP from a motor neuron specific GAL4-driver and co-stained with anti-Cbp53E antibodies. While the expression patterns were not identical, high magnification of single slice images revealed significant colocalization of GFP and Cbp53E in subsets of cells (Fig 2B. Pearson’s coefficient = 0.993). Strong Cbp53E expression was also visualized exiting the central nervous system in nearly every nerve bundle (Fig 2C). We therefore followed the nerves out to the peripheral nervous system to determine potential synaptically localized Cbp53E.

**Fig 3:**
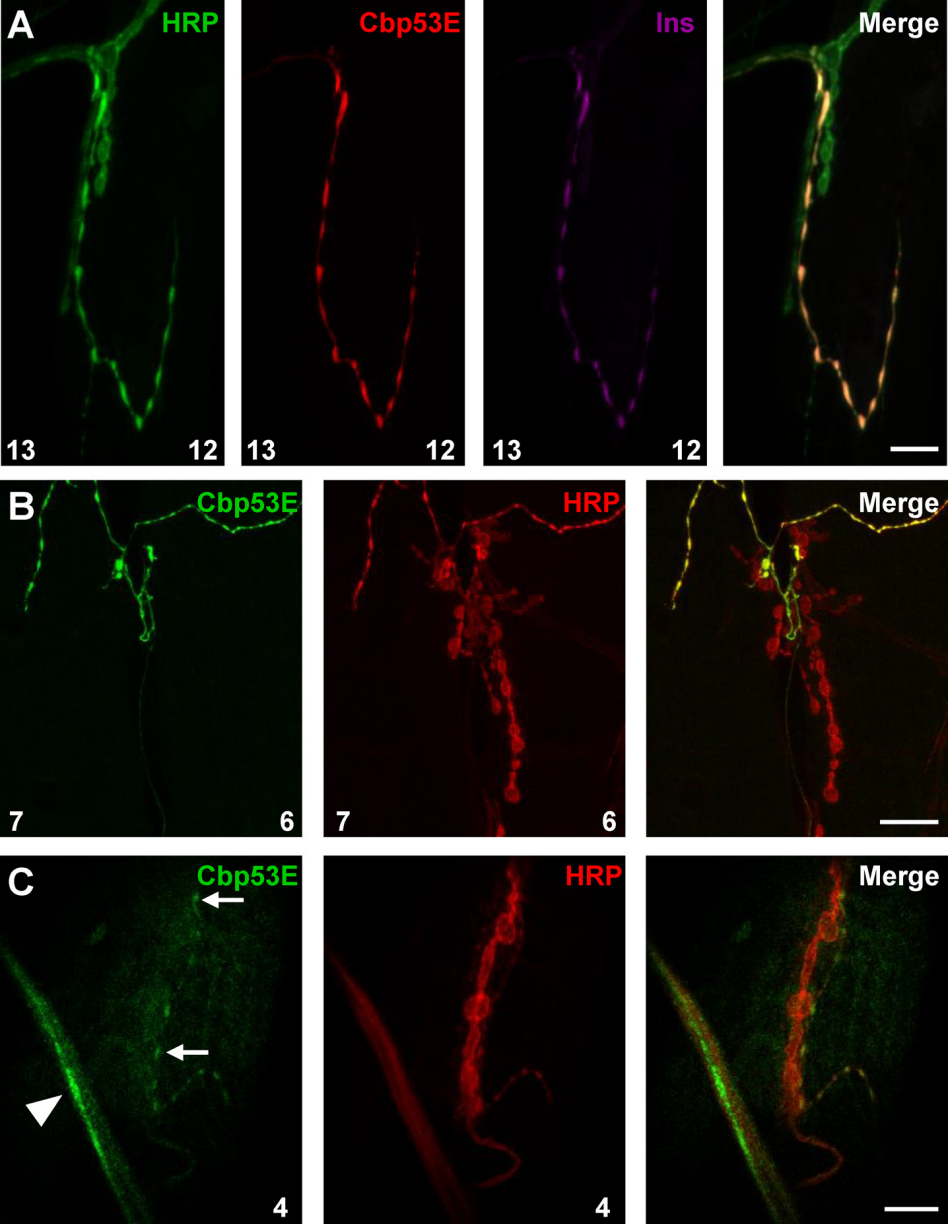
Expression of Cbp53E at the larval neuromuscular junction. A) Insulin peptide (purple) and Cbp53E (red) co-label type III boutons on muscle 12 of the NMJ. HRP (green) labels all neurons. B) A representative maximum intensity projection of strong Cbp53E staining (green) in type II boutons on muscles 6/7. C) A representative single slice image of weak Cbp53E staining (green) in type II boutons (arrow) on muscle 4. Cbp53E can also be seen in the nerve fibers around most muscles (arrowhead). Scale bars in all panels are 20μm.

The larval neuromuscular junction (NMJ) is comprised of axons innervating muscles and forming multiple types of synaptic boutons which can be characterized by their size and neurotransmitter properties. Type Ib and Is boutons are primarily glutamatergic, while type II and type III boutons are peptidergic [40–43]. The strongest Cbp53E expression at the NMJ was seen in type II and type III synaptic boutons. Type II boutons are readily identified by their small, bead-like morphology, but type III boutons can only be conclusively characterized by the presence of insulin [41]. We therefore co-stained tissues with the neuronal marker anti-HRP, anti-Cbp53E, and anti-insulin antibodies and observed strong overlap of Cbp53E and insulin on muscle 12 in control animals (Fig 3A). Notably, *Cbp53E* ^*Mi22*^ animals showed no Cbp53E NMJ localization (S2 Fig). Expression of Cbp53E in type II boutons, however, was more variable. We could find many instances of strong Cbp53E expression in type II boutons on many muscles including muscles 4, 6/7 and 12/13. One example of strong expression is shown on muscles 6/7 in Fig 3B. Sometimes, however, expression in type II boutons was very weak as seen in the type II innervation present on muscle 4 (Fig 3C). There was no obvious pattern to this discrepancy in expression levels. Often times, type II boutons on different muscles in the same abdominal segment stained differently for Cbp53E. Furthermore, we examined NMJs of muscles 4, 6/7, and 12/13 and could not find any expression of Cbp53E in type I glutamatergic synapses. Thus, levels of Cbp53E that are detectable by immunohistochemistry seem to be associated specifically with peptidergic synapses at the NMJ.

### *Drosophila* Cbp53E regulates neuronal development

**Fig 4:**
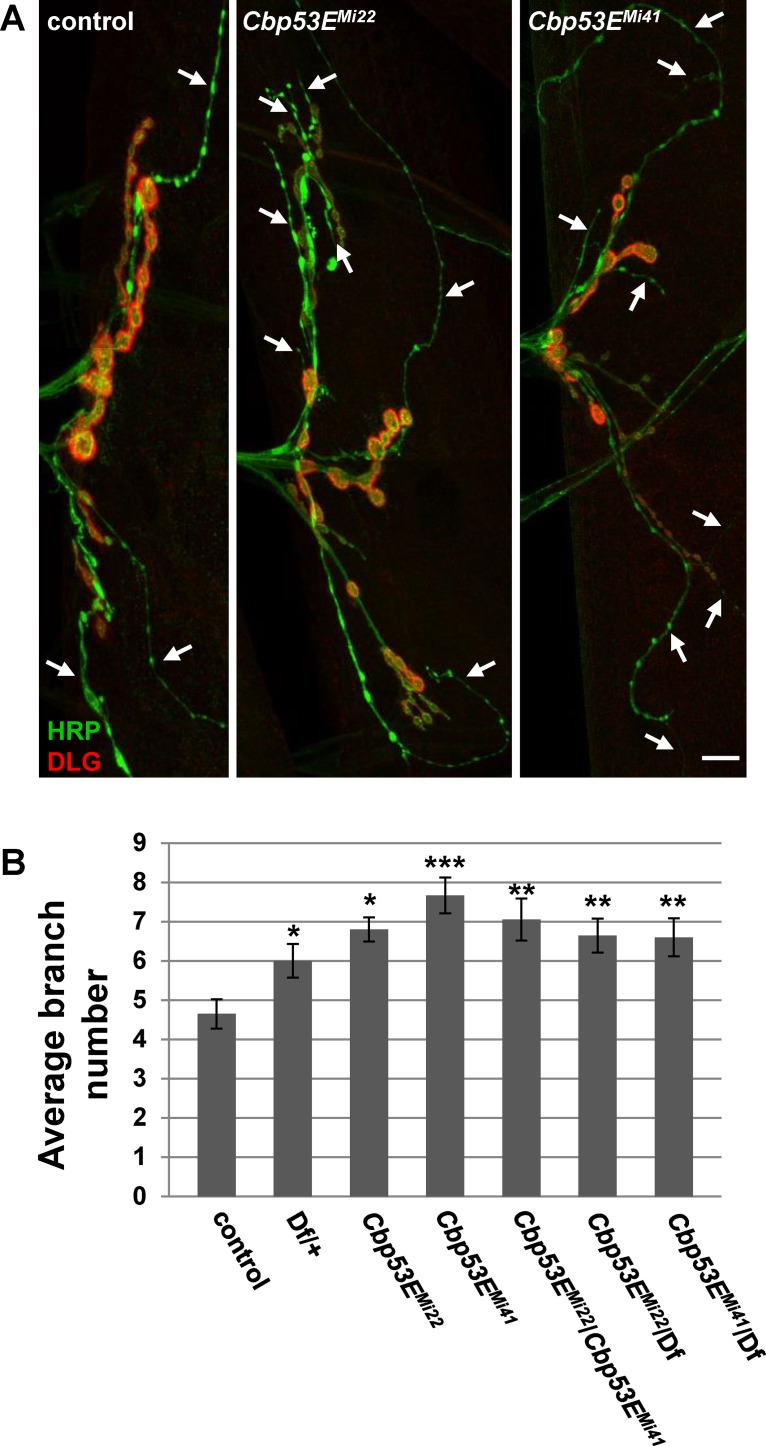
Loss of Cbp53E increases axon branching of type II/III terminals. A) Representative maximum intensity projections of muscle 12 innervations. All synapses are labeled with anti-HRP (green) while type I synapses are eliminated from analysis with anti-DLG labelling (red). Arrows point to type II/III axons branches of the indicated genotypes. Scale bar is 10μm. B) Quantitation of the average branch number of type II/III innervations from the indicated genotypes. * 0.01<p<0.05, ** 0.001<p<0.01, ***p<0.001.

The *Drosophila* larval neuromuscular junction is a well-characterized model of neuronal development [44]. Since Cbp53E is localized to at least one class of NMJs we wanted to determine whether loss of function of Cbp53E could alter the growth of the NMJ. Since Cbp53E is most strongly expressed in type II and type III boutons on muscle 12, we combined the analysis of these innervations in control and multiple mutant lines. No reproducible differences in the number of type II/III synaptic boutons between control animals and either *Cbp53E* ^*Mi22*^ or *Cbp53E* ^*Mi41*^mutants could be identified (data not shown). We therefore focused on quantifying the axon branching patterns of type II/III innervations. Both *Cbp53E* ^*Mi22*^ and *Cbp53E* ^*Mi41*^mutant alleles showed an increase in type II/III neuronal branching relative to controls (Fig 4. control: 4.7 +/- 0.37, n = 10; *Cbp53E* ^*Mi22*^: 6.8 +/- 0.31, n = 10, vs. control: p<0.05; *Cbp53E* ^*Mi41*^: 7.7 +/- 0.46, n = 9, vs. control: p<0.001). This finding remained consistent in the *Cbp53E* ^*Mi22*^/*Cbp53E* ^*Mi41*^ trans-heterozygote (*Cbp53E* ^*Mi22*^/*Cbp53E* ^*Mi41*^: 7.1 +/-0.54, n = 9, vs. control: p<0.01). While a heterozygote deficiency line spanning the Cbp53E genomic locus exhibited a slight increase in branching, each Cbp53E allele in combination with the deficiency produced more significant increases in branch number (Df/+: 6.0 +/- 0.43, n = 12, vs. control: p<0.05; *Cbp53E* ^*Mi22*^/Df: 6.6 +/- 0.43, n = 14, vs. control: p<0.01; *Cbp53E* ^*Mi41*^/Df: 6.6 +/- 0.48, n = 10, vs. control: p<0.01). None of the mutant allele combinations were significantly different than the heterozygote deficiency line alone (all comparisons p>0.05) which is likely due to the large deletion affecting many genes. Thus, Cbp53E mitigates the extent of type II/III axonal arborization at the neuromuscular junction.

**Fig 5:**
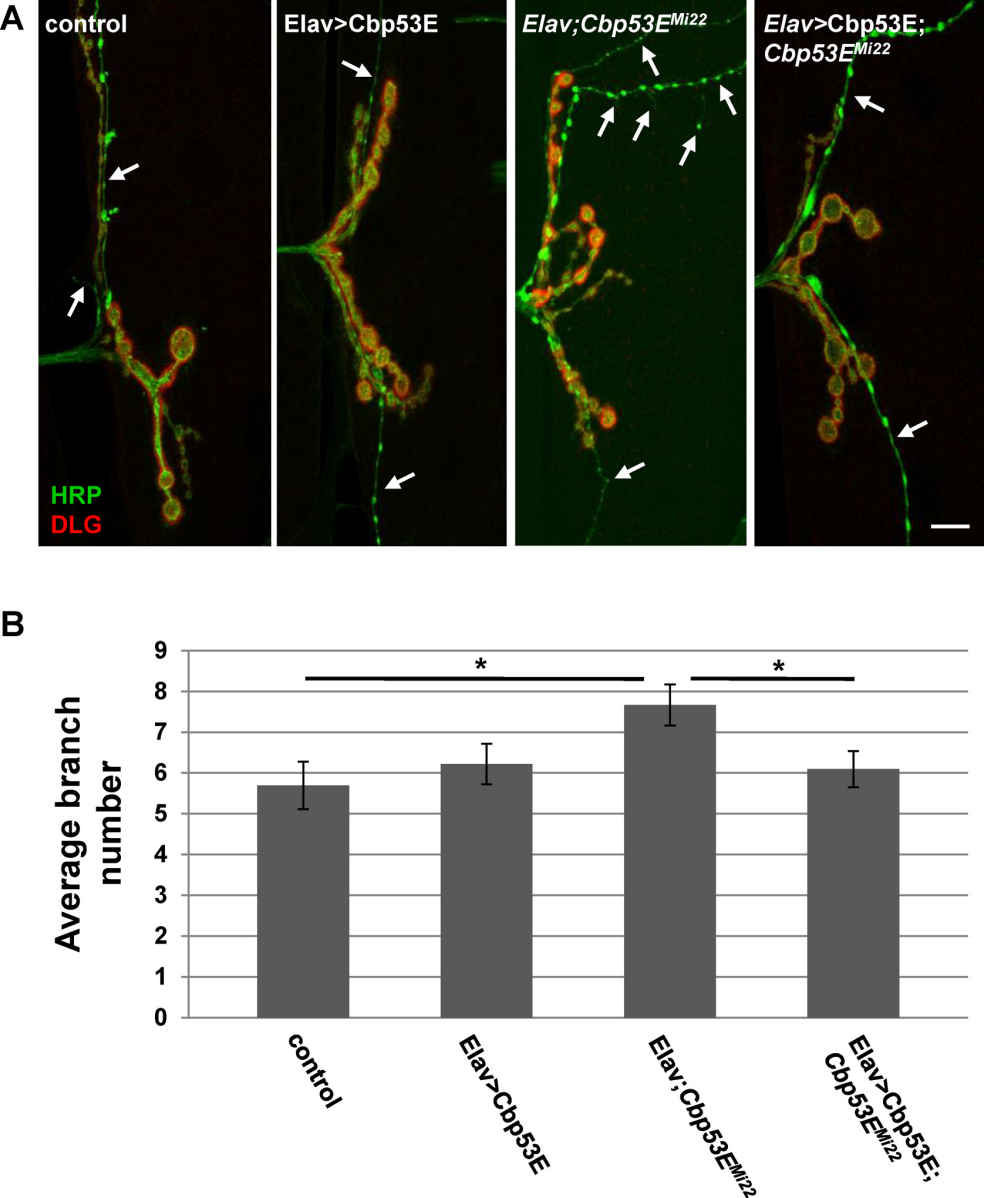
Neuronal expression of transgenic Cbp53E rescues type II/III axon branching defects. A) Representative maximum intensity projections of muscle 12 innervations labeled with anti-HRP (green) and anti-DLG (red). Arrows point to type II/III axon branches of the indicated genotypes. Scale bar is 10μm. B) Quantitation of the average branch number of type II/III innervations from the indicated genotypes. *0.01<p<0.05.

**Fig 6:**
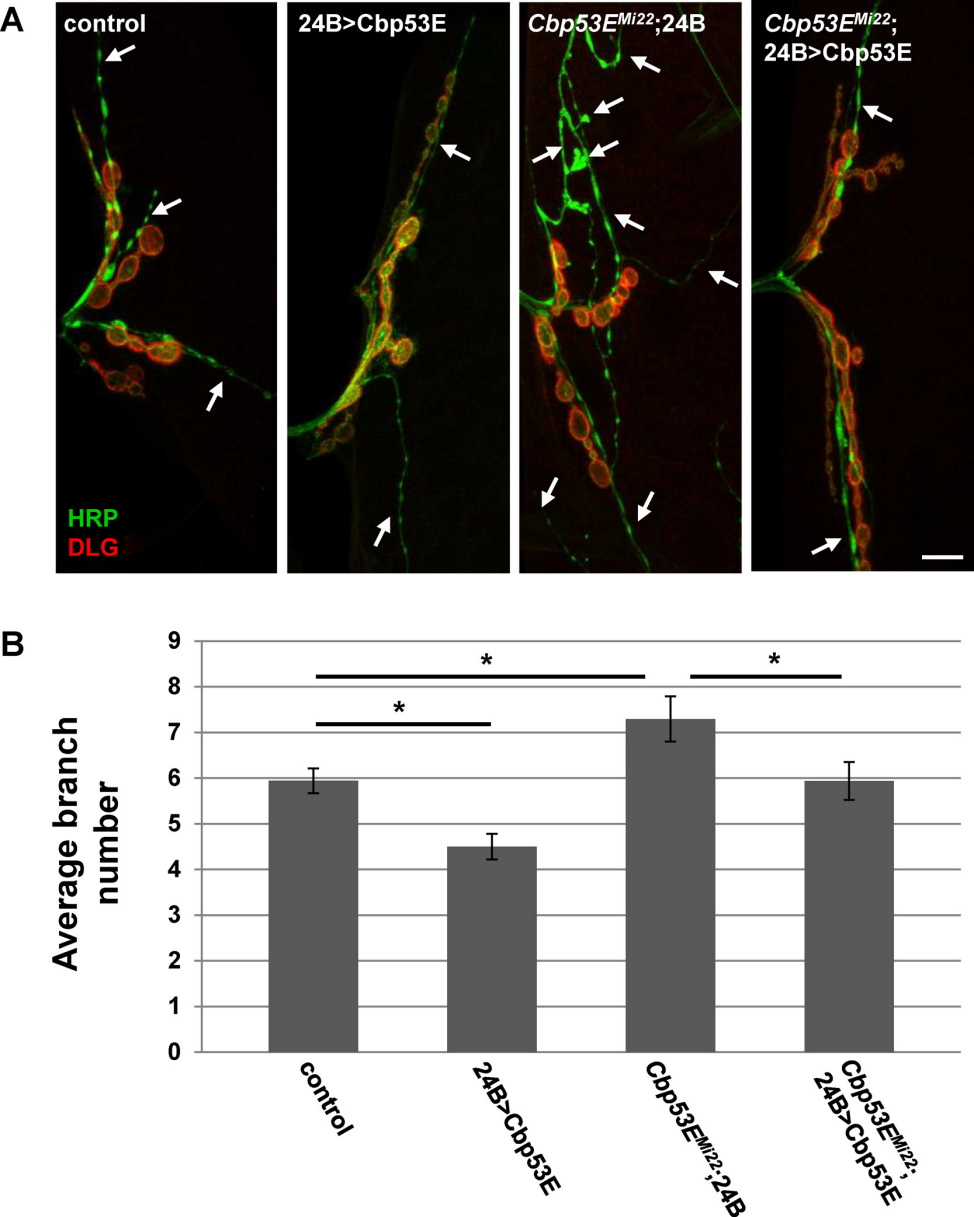
Muscle expression of transgenic Cbp53E rescues type II/III axon branching defects. A) Representative maximum intensity projections of muscle 12 innervations labeled with anti-HRP (green) and anti-DLG (red). Arrows point to type II/III axon branches of the indicated genotypes. Scale bar is 10μm. B) Quantitation of the average branch number of type II/III innervations from the indicated genotypes. *0.01<p<0.05.

Since loss of function of Cbp53E resulted in an increase in axonal branching, we also examined the effects of Cbp53E overexpression on neuronal development. We constructed a UAS-driven Cbp53E transgene and expressed it presynaptically with an elav-GAL4 driver. Surprisingly, there was no difference in type II/III axonal branches between controls and Cbp53E neuronal overexpression animals (Fig 5. control: 5.7 +/- 0.58, n = 18; Elav>Cbp53E: 6.2 +/-0.5, n = 16, vs. control: p>0.05). This construct was functional in neurons, however, as elav-GAL4 directed expression of Cbp53E in the *Cbp53E* ^*Mi22*^ null background completely rescued the type II/III axon overgrowth (Elav/+; *Cbp53E* ^*Mi22*^: 7.7 +/- 0.51, n = 15, vs. control: p<0.05; Elav>Cbp53E; *Cbp53E* ^*Mi22*^: 6.1 +/- 0.44, n = 16, vs Elav/+; *Cbp53E* ^*Mi22*^: p<0.05). We also expressed Cbp53E postsynaptically at the NMJ with the muscle specific driver 24B-GAL4 (Fig 6). In addition to rescuing the overgrowth seen in *Cbp53E* ^*Mi22*^ neurons, postsynaptic expression of Cbp53E alone resulted in a decrease in type II/III axonal branching (control: 5.9 +/- 0.27, n = 17; 24B>Cbp53E: 4.5 +/- 0.28, n = 13, vs. control: p<0.05, *Cbp53E* ^*Mi22*^;24B/+: 7.3 +/- 0.5, n = 17, vs. control: p<0.05; *Cbp53E* ^*Mi22*^;24B>Cbp53E: 5.9 +/- 0.42, n = 16, vs. *Cbp53E* ^*Mi22*^;24B/+: p<0.05). To ensure that rescue of branching defects in the Cbp53E nulls was not due to a potentially leaky transgene, we compared the number of branches from each driver in the mutant background with the transgene in the mutant background and could not detect any differences (*Cbp53E* ^*Mi22*^;24B/+: 7.3 +/- 0.5, n = 17; Elav/+; *Cbp53E* ^*Mi22*^: 7.7 +/- 0.51, n = 15; Cbp53E^Mi22^; Cbp53E/+: 7.2 +/- 0.4, n = 17. ANOVA p = 0.79). Thus, neuronal growth defects stemming from genetic loss of function alleles of Cbp53E can be recovered by either pre- or postsynaptic introduction of transgenic Cbp53E. However, only the postsynaptic compartment at the NMJ is sensitive to excessive Cbp53E expression with respect to type II/III innervations.

**Fig 7:**
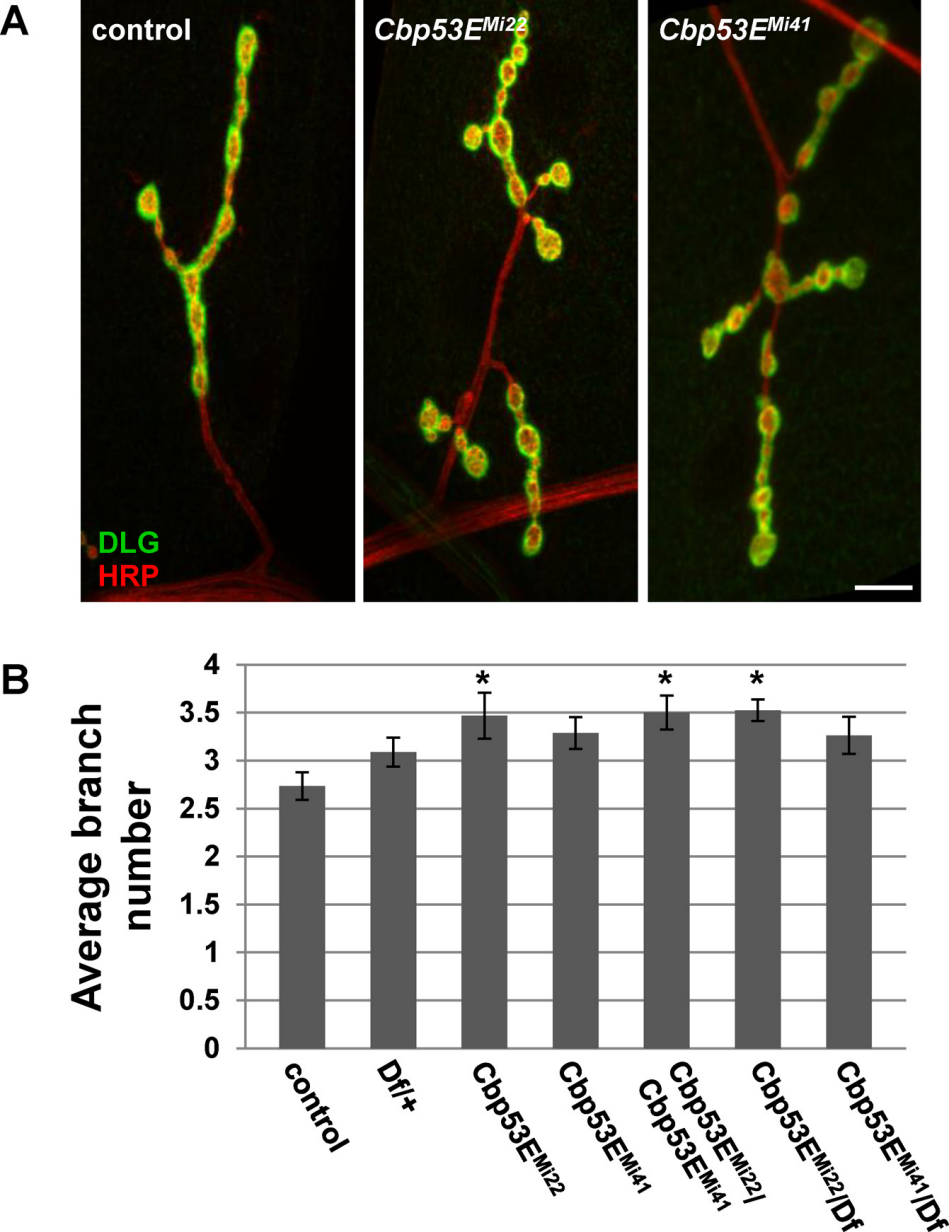
Loss of Cbp53E increases type Ib axonal branching at the NMJ. A) Representative maximum intensity projections of muscle 4 type Ib innervations from the indicated genotypes stained with anti-DLG (green) and anti-HRP (red). Scale bar is 10μm. B) Quantitation of the average branch number of type Ib innervations from the indicated genotypes. * 0.01<p<0.05.

Even though we could not detect Cbp53E expression in type I glutamatergic synapses, we wanted to determine if these synapses were also affected by loss of function of Cbp53E. We therefore quantified type Ib innervations at the well-characterized larval muscle 4. This glutamatergic synaptic arborization has a simple, easily-quantified pattern of innervation branches and boutons and has frequently been used as a model for glutamatergic neuronal development [45–48]. As with type II/III boutons on muscle 12, we were unable to identify any changes in type Ib bouton number between control animals and Cbp53E mutant alleles. We therefore quantified type Ib axon branch numbers in controls and all mutant allele combinations as before. Only the *Cbp53E* ^*Mi22*^ null mutant animals showed a significant increase in the average number of axon branches within the muscle 4 arbor (Fig 7. control: 2.7 +/- 0.14, n = 17; *Cbp53E* ^*Mi22*^: 3.5 +/- 0.24, n = 16, vs. control: p<0.05; *Cbp53E* ^*Mi41*^: 3.3 +/- 0.17, n = 14, vs. control: p>0.05). The excessive branching seen in *Cbp53E* ^*Mi22*^ was, however, present in all trans-heterozygote combinations, though not in the deficiency heterozygote line alone (Df/+: 3.1 +/- 0.15, n = 17, vs. control: p>0.05; *Cbp53E* ^*Mi22*^/*Cbp53E* ^*Mi41*^: 3.5 +/-0.18, n = 16, vs. control: p<0.05; *Cbp53E* ^*Mi22*^/Df: 3.5 +/- 0.11, n = 20, vs. control: p<0.05). As seen at type II/III NMJs, none of the allelic combinations were significantly different from the heterozygote deficiency line (p>0.05). Thus, we conclude that the *Cbp53E* ^*Mi41*^ allele is somewhat weaker than the *Cbp53E* ^*Mi22*^ allele. Nevertheless, in addition to regulating peptidergic terminal formation, Cbp53E also seems to mitigate the extent of glutamatergic axonal growth at the neuromuscular junction.

**Fig 8:**
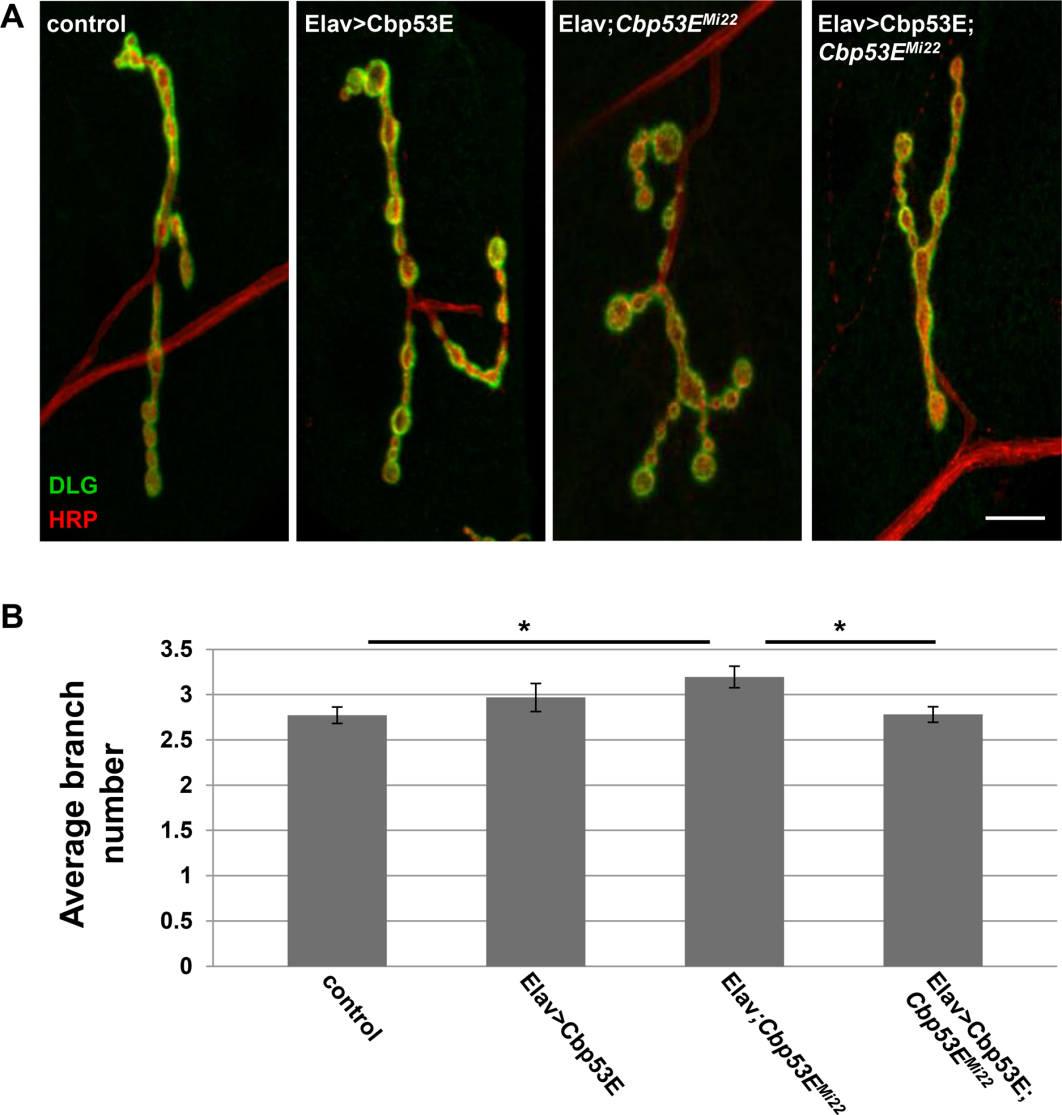
Neuronal expression of transgenic Cbp53E rescues type Ib axon branching defects. A) Representative maximum intensity projections of muscle 4 type Ib innervations from the indicated genotypes stained with anti-DLG (green) and anti-HRP (red). Scale bar is 10μm. B) Quantitation of the average branch number of type Ib innervations from the indicated genotypes. *0.01<p<0.05.

**Fig 9:**
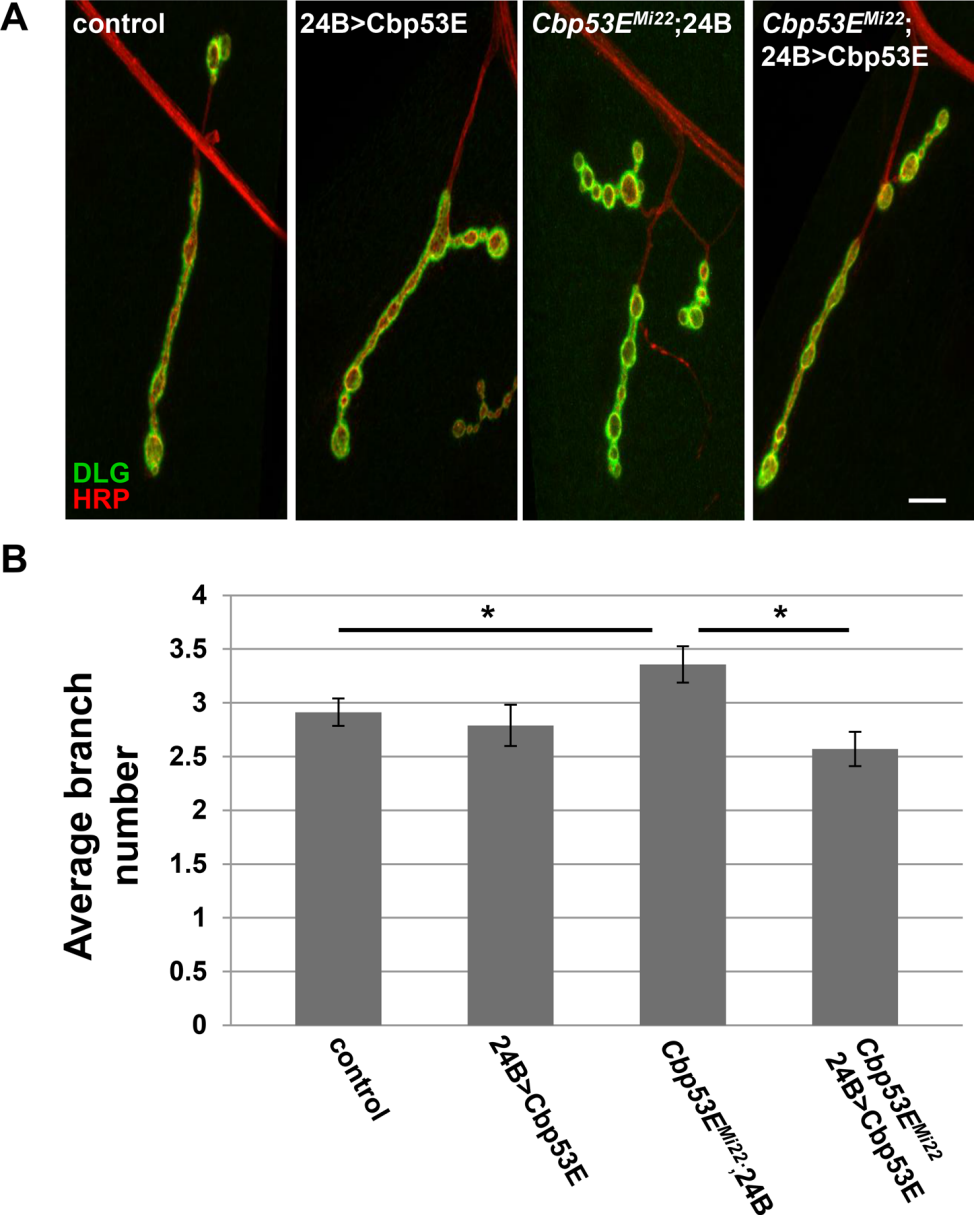
Muscle expression of transgenic Cbp53E rescues type Ib axon branching defects. A) Representative maximum intensity projections of muscle 12 innervations labeled with anti-DLG (green) and anti-HRP (red). Scale bar is 10μm. B) Quantitation of the average branch number of type Ib innervations from the indicated genotypes. * 0.01<p<0.05.

We next characterized the effects of both pre- and postsynaptic overexpression of Cbp53E on muscle 4 type Ib axon branches. Surprisingly, unlike type II/III innervations on muscle 12, there were no differences detected in branch number of type Ib arbors between control and either Cbp53E overexpression condition (Figs 8 and 9) (Neuronal expression: control: 2.8 +/- 0.09, n = 22; Elav>Cbp53E: 3.0 +/- 0.15, n = 16, vs. control: p>0.05. Muscle expression: control: 2.9 +/- 0.13, n = 23; 24B>Cbp53E: 2.8 +/-0.19, n = 19, vs. control: p>0.05). However, as was seen in peptidergic terminals, Cbp53E can function both pre-and postsynaptically as evidenced by the targeted ability to rescue the *Cbp53E* ^*Mi22*^ null overgrowth (Elav/+; *Cbp53E* ^*mi22*^: 3.2 +/- 0.12, n = 28 vs. control: p<0.05; Elav>Cbp53E; *Cbp53E* ^*Mi22*^: 2.8 +/- 0.09, n = 32, vs. Elav/+; *Cbp53E* ^*mi22*^: p<0.05; *Cbp53E* ^*Mi22*^;24B/+: 3.2 +/-0.17, n = 21, vs. control: p<0.05; *Cbp53E* ^*Mi22*^;24B>Cbp53E: 2.6 +/- 0.16, n = 20, vs. *Cbp53E* ^*Mi22*^;24B/+: p<0.05). As with type II/III NMJs we did not detect any difference between the driver lines in the mutant background and the transgene in the mutant background indicating that this rescue is likely not the result of a leaky transgene (Elav/+; *Cbp53E* ^*mi22*^: 3.2 +/- 0.12, n = 28; *Cbp53E* ^*Mi22*^;24B/+: 3.2 +/-0.17, n = 21; *Cbp53E* ^*Mi22*^; Cbp53E/+ 3.5 +/- 0.3, n = 17. ANOVA p = 0.4). Thus, loss of function of Cbp53E results in an increase in axon branching of glutamatergic innervations, which can be rescued by expressing Cbp53E either in neurons or in the muscle. However, irrespective of cellular compartment, overexpression of Cbp53E alone does not seem to affect glutamatergic neuronal development.

## Discussion

For over 100 years, *Drosophila* have been a key model organism in understanding genes and pathways involved in organismal development. Due to the strong evolutionary conservation of many genes, researchers are now turning to *Drosophila* to mimic the genetic alterations found in human diseases, including neurodevelopmental disorders such as Rett Syndrome and Angelman Syndrome and neurodegenerative disorders such as Parkinson’s disease and Alzheimer’s disease[32, 49, 50]. Despite this increased effort to exploit *Drosophila* for translational research, little attention has been given to the critical contributions of calcium regulating genes in these models. Since calcium is essential for nearly all cellular functions, we began bridging this gap by investigating the function of the fly’s only 6 EF-hand domain containing protein, Cbp53E[33]. Cbp53E null mutant alleles were generated and then used in neurodevelopmental studies (Fig 1). Cbp53E null animals are homozygous viable and show no overt abnormalities, similar to the knockout animals of the vertebrate family of 6 EF-hand domain containing proteins [21, 22].

We confirmed previously identified discrete expression of Cbp53E in the larval neuropil and further identified that this expression resides in a subset of motor neurons (Fig 2) [33]. This is consistent with the selective expression of CB/CR throughout the vertebrate nervous system, including in many lower motor neurons [51]. In adult *Drosophila*, Cbp53E is also expressed in some muscles and some neurons innervating flight muscles [33]. At the larval NMJ, we could not detect Cbp53E expression in muscles, however we did identify strong expression in peptidergic type II and type III synaptic innervations (Fig 3). This finding prompted us to analyze neuronal architecture at the NMJ in Cbp53E mutants.

*Cbp53E* null mutants exhibit overgrowth of terminal axon branches in both peptidergic and glutamatergic synapses (Figs 4 and 7). It is therefore possible that the excessive branching at two different classes of synaptic terminals occurs via two distinct mechanisms. Cbp53E is localized to type II/III synaptic boutons and therefore may participate directly in synaptic mechanisms regulating neuronal growth. However, Cbp53E does not appear to be expressed at type I larval NMJs (Fig 3). While we cannot rule out that Cbp53E is expressed at type I synapses below the levels of our detection, since loss of function of Cbp53E still affects type I NMJ growth, a more global, rather than synaptic mechanism may direct events at these synapses. Alternatively, it is possible that localization of Cbp53E to type II/III synapses is not at all directly related to its function in neuronal growth but serves some other uncharacterized purpose. In this scenario, somatic control of axon growth by Cbp53E could govern both peptidergic and glutamatergic arbor development. Further experiments directed at separating soma and synaptic mechanisms, however, are required to distinguish between these possibilities.

Even though we could not detect Cbp53E endogenously in the the larval muscle, it is interesting that both pre- and postsynaptic expression of Cbp53E is able to rescue all axonal branching defects in Cbp53E null animals. One important caveat to these findings is that while the 24B-gal4 driver used in this study is often used to target transgene expression in the muscle, it does also express transgenes in some neurons. We examined this expression pattern in the larval system, however, and could not find any overlap between neurons which express 24B-gal4 and endogenous Cbp53E either in cell soma, or at the NMJs examined in this study (S3 Fig). Nevertheless, it remains possible that neurons expressing transgenic Cbp53E from the 24B-gal4 driver could produce some circuit based effects which alter the axon development of the motor neurons studied here.

Somewhat mitigating this interpretation, however, is the finding that postsynaptic muscle overexpression of Cbp53E is able to reduce axon branching of type II/III terminals while presynaptic overexpression has no effect (Figs 5 and 6). This result would suggest that any effect of presynaptic Cbp53E expression from the 24B-gal4 driver is offset by Cbp53E being present in the muscle. It is unclear why reduction of axon branch elaboration is limited to a postsynaptic function of Cbp53E. It is possible that there is a level of functional Cbp53E saturation that is already met by endogenous Cbp53E in neurons. Since muscles do not express appreciable levels of Cbp53E, this hypothetical threshold is not met in muscles thereby allowing transgenic Cbp53E to alter axon growth. The mechanism of this apparent retrograde function remains to be determined.

It is also interesting that peptidergic type II/III terminals are more sensitive than the type I glutamatergic terminals to levels of Cbp53E. Specifically, the effect of Cbp53E overexpression on axon growth does not occur at type I NMJs. Since Cbp53E is expressed at type II/III synapses but not type I synapses, it is possible that this result does in fact point to a specific synaptic control mechanism of axon growth that is dependent on Cbp53E. However, the differences in growth effects on these terminals could also be due to the inherent functional differences of the synapses. It is known for example that the neurexin/neuroligin system modulates in order to differentially regulate the formation of different classes of synapses [52]. Differential regulation of these molecules in the context of Cbp53E null animals could potentially explain why overexpression of Cbp53E uniquely affects type I and type II/III NMJs. More functional characterization of Cbp53E in these pre- and postsynaptic contexts will be necessary to elucidate the exact mechanism of these axon growth defects.

We have used the *Drosophila* NMJ as a model of neuronal growth to begin to understand the role of Cbp53E in the developing nervous system. Though data reported here support the hypothesis that Cbp53E regulates neuronal development, it is clear that many questions still remain about Cbp53E function. The CB/CR family of calcium binding proteins possesses well-characterized calcium buffering properties, however the calcium buffering capacity of Cbp53E remains to be empirically determined. Nevertheless, the animals we have described can now be used to investigate the diverse contributions of the CB/CR proteins in *Drosophila* genetic models of human diseases. In particular, it will be interesting to determine whether this single gene can provide the neuroprotective functions of the mammalian family in degenerative disorders such as PD and AD. Incorporating genetic alterations of Cbp53E will surely enhance the value of these and other models and potentially uncover important calcium-dependent mechanisms underlying each disease.

## Supporting Information

S1 Fig Expression of Cbp53E is absent in *Cbp53E* ^*Mi22*^ larval brains. Control (A) and *Cbp53E* ^*Mi22*^ (B) larval brains were stained with anti-Cbp53E and anti-HRP antibodies and then imaged under identical conditions. Scale bar is 10μm.
(TIF)

S2 Fig Expression of Cbp53E is absent in *Cbp53E* ^*Mi22*^ muscle 12 NMJs. Control (A) and *Cbp53E* ^*Mi22*^ (B) larval segment 3 muscle 12 NMJs were stained with anti-Cbp53E and anti-HRP antibodies and then imaged under identical conditions. Scale bar is 10μm.
(TIF)

S3 Fig 24B-gal4 drives expression in some neurons but is postsynaptic at the NMJ. (A) Larval brains from animals using 24B-gal4 to drive expression of mCD8GFP were stained with anti-Cbp53E antibodies. Each slice panel is a single representative optical section from 3 different depths of the same brain. The final panel is a maximum intensity projection of the image stack. Arrows indicate neurons expressing GFP from the 24B-gal4 driver. We could not find any cells expressing both GFP and Cbp53E. (B) Muscle 4 NMJ from the animals in (A) stained with HRP to denote the presynaptic compartment and DLG to denote the postsynaptic compartment. GFP overlaps only with DLG. (C) Muscle 12 NMJ from the animals in (A) stained with anti-Cbp53E antibodies. GFP does not appear in type II/III synapses containing Cbp53E, thus indicating only postsynaptic expression at this synapse. Scale bars are 10μm.
(TIF)
