# Supplementary figures and images for: Drosophila Cbp53E Regulates Axon Growth at the Neuromuscular Junction
Source: PLoS One. 2015 Jul 13;10(7):e0132636. doi: 10.1371/journal.pone.0132636 (PMC4500412; doi:10.1371/journal.pone.0132636)

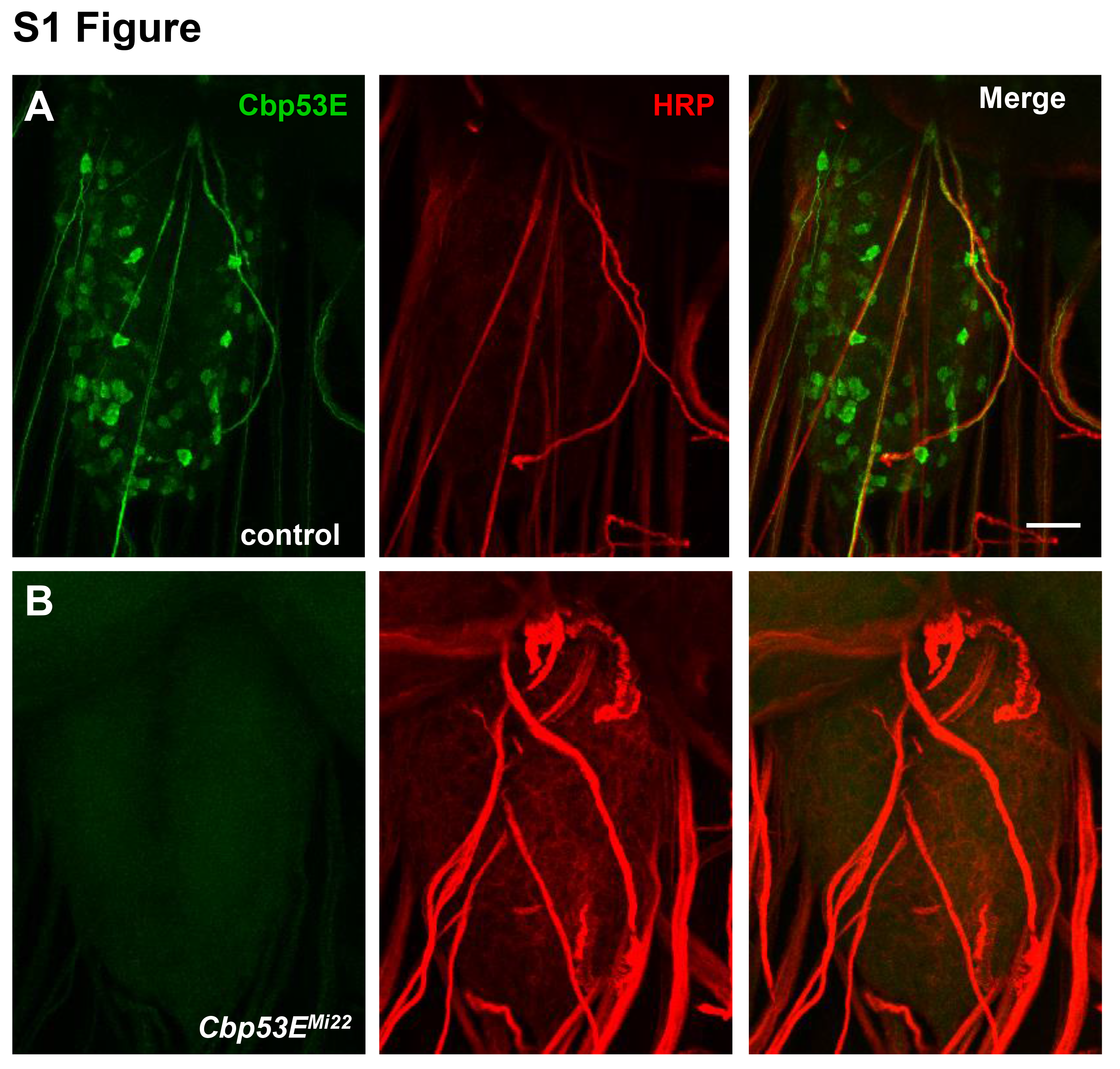

Supplement: S1 Fig — Control (A) and Cbp53E Mi22 (B) larval brains were stained with anti-Cbp53E and anti-HRP antibodies and then imaged under identical conditions. Scale bar is 10μm. (TIF) [file pone.0132636.s001.tif]

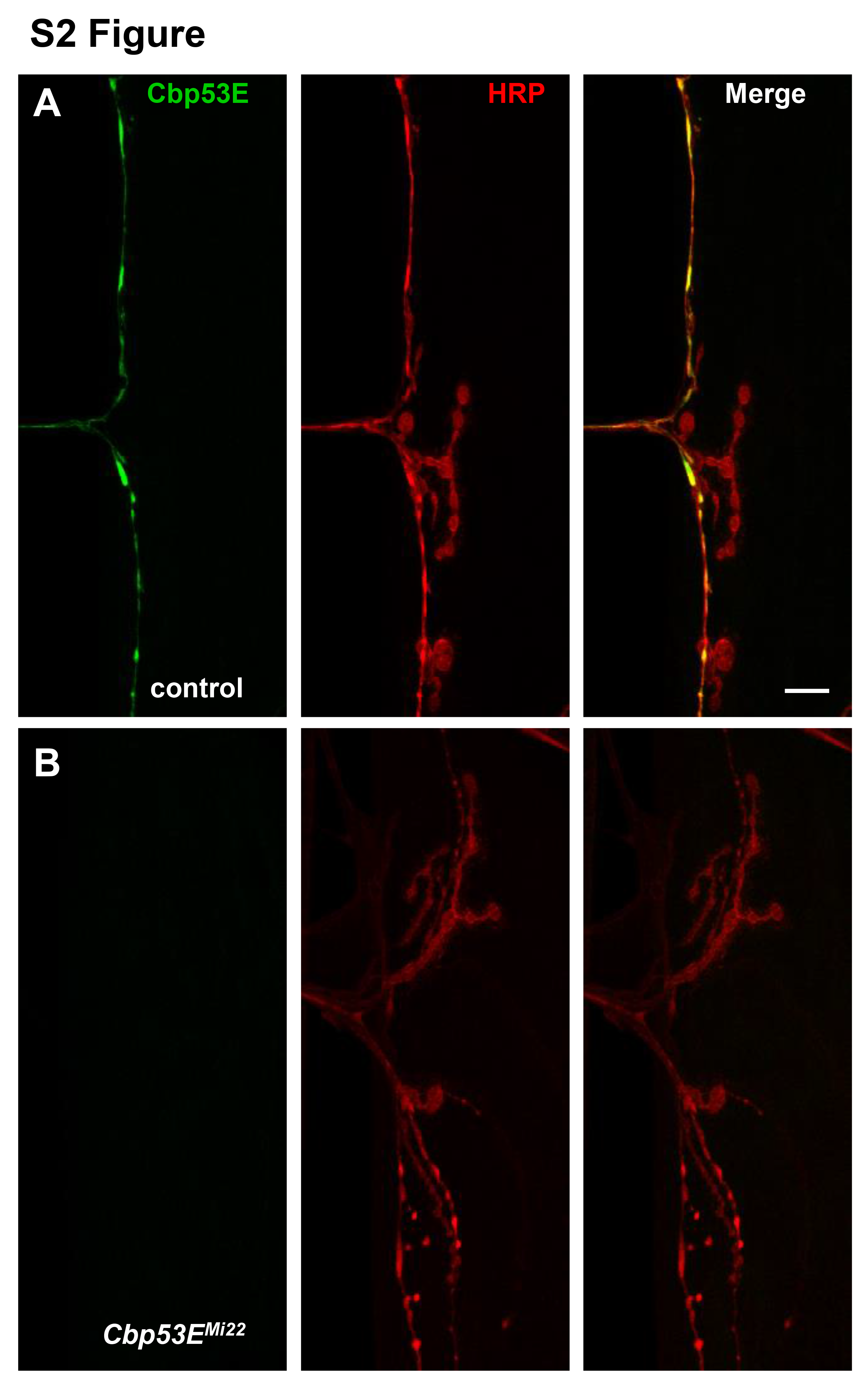

Supplement: S2 Fig — Control (A) and Cbp53E Mi22 (B) larval segment 3 muscle 12 NMJs were stained with anti-Cbp53E and anti-HRP antibodies and then imaged under identical conditions. Scale bar is 10μm. (TIF) [file pone.0132636.s002.tif]

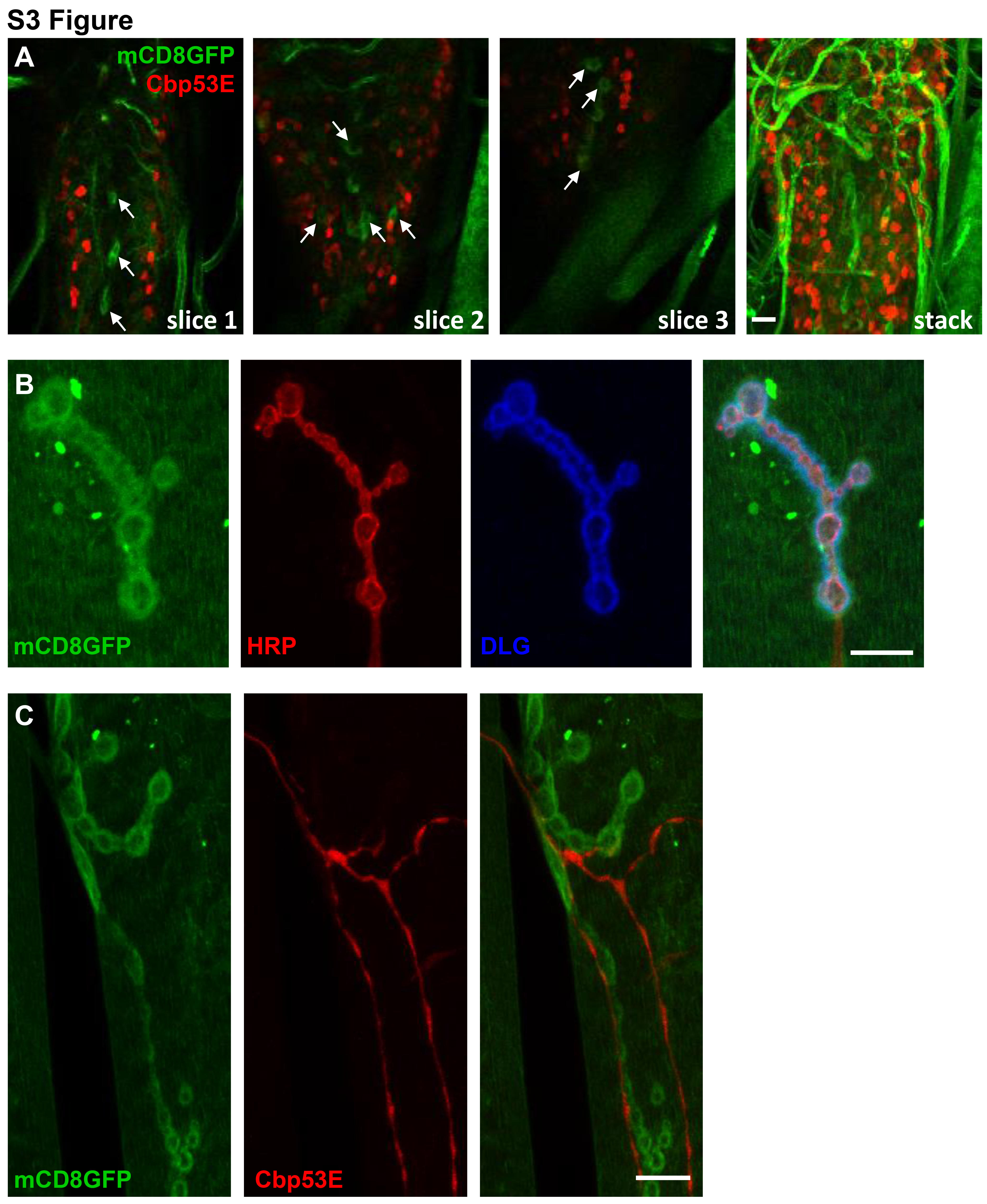

Supplement: S3 Fig — (A) Larval brains from animals using 24B-gal4 to drive expression of mCD8GFP were stained with anti-Cbp53E antibodies. Each slice panel is a single representative optical section from 3 different depths of the same brain. The final panel is a maximum intensity projection of the image stack. Arrows indicate neurons expressing GFP from the 24B-gal4 driver. We could not find any cells expressing both GFP and Cbp53E. (B) Muscle 4 NMJ from the animals in (A) stained with HRP to denote the presynaptic compartment and DLG to denote the postsynaptic compartment. GFP overlaps only with DLG. (C) Muscle 12 NMJ from the animals in (A) stained with anti-Cbp53E antibodies. GFP does not appear in type II/III synapses containing Cbp53E, thus indicating only postsynaptic expression at this synapse. Scale bars are 10μm. (TIF) [file pone.0132636.s003.tif]
